# RNF146 Inhibits Excessive Autophagy by Modulating the Wnt-β-Catenin Pathway in Glutamate Excitotoxicity Injury

**DOI:** 10.3389/fncel.2017.00059

**Published:** 2017-03-06

**Authors:** Yuefan Yang, Peng Luo, Haoxiang Xu, Shuhui Dai, Wei Rao, Cheng Peng, Wenke Ma, Jiu Wang, Hongyu Xu, Lei Zhang, Sai Zhang, Zhou Fei

**Affiliations:** ^1^Department of Neurosurgery, Xijing Hospital, Fourth Military Medical UniversityXi’an, China; ^2^Department of Biomedical Engineering, Fourth Military Medical UniversityXi’an, China; ^3^Department of Neurosurgery, 411 Hospital of People’s Liberation ArmyShanghai, China; ^4^Department of Neurosurgery, Affiliated Hospital of Logistics, University of Chinese Armed Police ForcesTianjin, China

**Keywords:** glutamate, excitotoxicity, RNF146, autophagy, Wnt/β-catenin signaling

## Abstract

Glutamate induced excitotoxicity is common in diverse neurological disorders. RNF146 as an E3 ubiquitin ligase protects neurons against excitotoxicity *via* interfering with Poly (ADP-ribose) (PAR) polymer-induced cell death (parthanatos). However, the neuroprotective role of RNF146 has not been fully understood. We aimed to investigate the role of RNF146 in modulating autophagy in HT22 cells under glutamate excitotoxicity injury. Here we found that induction of RNF146 decreased the cellular damage and excitotoxicity induced by glutamate. RNF146 also suppressed the excessive autophagy, which is detrimental to HT22 cells survival, induced by glutamate or rapamycin treatment. In addition, we find that Wnt/β-catenin was a negative regulation factor for autophagy in glutamate excitotoxicity. Over-expression of RNF146 promoted Wnt/β-catenin signaling, which was related to destabilization of β-catenin destruction complex. These results indicated that RNF146 acted as a neuroprotective agent against glutamate-induced excitatory damage, and this neuroprotection might be at least partly dependent on the inhibition of excessive autophagy by regulating Wnt/β-catenin signaling.

## Introduction

Glutamate is an endogenous excitatory neurotransmitter in the central nervous system. Glutamate toxicity is a chief factor which contributes to neuronal cell death in stroke, traumatic brain injury (TBI), and neurodegenerative diseases, such as Parkinson’s and Alzheimer’s Disease (Arundine and Tymianski, 2004; Dong et al., 2009; Scott et al., 2011). Glutamate excitotoxicity could stimulate the dysfunction of calcium homeostasis and induce intracellular calcium overload via NMDA receptor. The elevated cytoplasmic calcium level may trigger mitochondria results in the production of reactive oxygen species (ROS; Castilho et al., 1999), induction of the permeability transition pore that leads to mitochondrial depolarization (Abramov and Duchen, 2008) and neuronal death (Stout et al., 1998). Autophagy is the major catabolic pathway that is involved in the degradation of damaged or impaired organelles, abnormal protein and other cytosolic components. It is also an important cellular activity following glutamate-induced excitotoxicity. Evidence from *in vitro* studies suggests that activation of autophagy via pharmacological activators, including rapamycin and trehalose, may counteract the effects of glutamate-induced excitotoxicity (Kulbe et al., 2014). On the contrary, inhibition of autophagy was also believed responsible for neuroprotective effects in glutamate-induced injury (Kim et al., 2009; Chen et al., 2010). Furthermore, 3-MA, Ly294002 (VPS34 inhibitor), carnosine (an endogenous pleiotropic dipeptide) and insulin-like growth factor 1 attenuated several autophagic markers and decreased the demise of cultured neurons exposed to NMDA or kainic acid (Sadasivan et al., 2010; Baek et al., 2014; Ginet et al., 2014; Wang et al., 2014; Galluzzi et al., 2016). Taken together, the authentic effect of autophagy on glutamate excitotoxicity remains to be elucidated.

RNF146, which is also called Iduna, is a Poly (ADP-ribose) (PAR)-dependent E3 ligase and a central regulatory molecule in PAR-polymerase-1 (PARP-1)-dependent cell death. Unlike apoptosis, necrosis, and autophagy, this novel subtype of cell death is defined as parthanatos (David et al., 2009). As an E3 ligase, RNF146 has been found to regulate the degradation of Axin2 through modulating Tankyrase, which is implicated in many important cellular functions, such as telomere homeostasis, mitosis, and vesicle trafficking. Previous studies have indicated that either constitutive or acute overexpression of RNF146 has a neuroprotective role *in vivo* (Andrabi et al., 2011). When compared with wild-type mice, RNF146-transgenic mice displayed enhanced neurological function after occlusion of the middle cerebral artery, which indicates that RNF146 protects against ischemia-induced neuronal injury (Andrabi et al., 2011). Moreover, our recent study suggests that RNF146 acts as a potential antioxidant by improving mitochondrial function and inhibiting oxidative stress-induced parthanatos, and these protective effects are dependent on the involvement of the ubiquitin-proteasome system (Xu et al., 2013). RNF146 is a positive regulator of Wnt signaling via mediating the tankyrase-dependent degradation of axin (Callow et al., 2011). It operates the ubiquitylation of tankyrase, axin and even its self. Depletion of RNF146 suppressed degradation of axin2, and the proteasome inhibitor MG132 blocked axin2 degradation (Zhang et al., 2011). Down regulation of RNF146 also decreases autocrine Wnt signaling in teratocarcinoma cells (Callow et al., 2011). Moreover, Gao observed that RNF146 probably modulated cyclinD1 and MMP7 by the Wnt/β-catenin signaling in lung cancer cells (Gao et al., 2014). As autophagy has several roles in differentiation and a major role of Wnt/β-catenin signaling is to block differentiation, there may exist a connection between Wnt/β-catenin signaling and autophagy. Recent studies have shown that Wnt/β-catenin signaling has a role in autophagy in prostate cancer cells (Lin et al., 2015) and WB-F344 cells (Xie et al., 2014). Petherick et al. (2013) indicated that β-catenin negatively modulates LC3 puncta and p62 expression in HT29 colorectal carcinoma cells and mouse intestinal epithelium *in vivo*. However, the involvement of this pathway in cell death caused by glutamate-induced excitotoxicity has not been reported. The role of RNF146 in glutamate-induced excitotoxicity and autophagy and its crosstalk with downstream signaling are still unclear.

Consequently, in this study, we investigated the effect of RNF146 on modulating autophagy to protect neuronal cells from glutamate-induced excitotoxicity and cell death and further elucidated the role of Wnt signaling in regulating autophagy and excitotoxicity.

## Materials and Methods

### Antibodies and Reagents

The primary antibody against RNF146 was obtained from NeuroMab (mouse anti RNF146, AB_10675284). Antibodies against LC3II (rabbit anti LC3, L8918), Beclin-1 (rabbit anti Beclin-1, SAB1306537) and P62 (rabbit anti P62, P0067) were obtained from Sigma-Aldrich. Antibodies against β-catenin (rabbit anti β-catenin, #8480) and β-actin (rabbit anti β-actin, #4970) were obtained from Cell signaling Technology. The secondary antibodies for immunostaining are Alexa 488 donkey anti-rabbit IgG and Alex 594 donkey anti-mouse IgG which were purchased from Abcam. Glutamate, Rapamycin, and chloroquine (CQ) were obtained from Sigma-Aldrich. IWP2, Foxy5 and JW74 were obtained from Tocris bioscience.

### Cell Culture

HT22 cells were obtained from the Institute of Biochemistry and Cell Biology, SIBS, CAS. The cells were grown in Dulbecco’s modified Eagle’s medium (Gibco) supplemented with 10% fetal bovine serum (HyClone Laboratories, Logan, UT, USA) and 1% antibiotics (penicillin/streptomycin). HT22 cells were seeded in 6-well culture dishes (10^6^ per well) and incubated until they reached 60%–70% confluency. Cells were transfected with lentiviruses for 4 h, then replaced with fresh medium and incubated for another 48 h. Rapamycin (4 μM) or CQ (10 uM) were added in transfection cells or normal cells for 2 h before glutamate (10 mM) treatment. Wnt inhibitor and agonist, JW74 (10 μM), IWP2 (10 μM) and Foxy5 (10 μM), were added 2 h before glutamate treatment. Following transfection and treatment with glutamate (10 mM) at different time points (2 h, 4 h, 8 h, 12 h, 24 h), the cells were subjected to various measurements.

### Western Blot Analysis

After different treatments, HT22 cells in 6 cm dishes were washed with ice-cold phosphate-buffered saline (PBS) for three times and lysed with a lysis buffer containing protease inhibitor mixture tablets and phosphatase inhibitor mixture tablets PhosSTOP (Roche Applied Science). The protein concentration of the supernatant was assessed by using a BCA protein kit. The proteins were separated by 10%–15% and 10% SDS-PAGE gels and transferred to nitrocellulose membranes (Invitrogen). The membranes were soaked in a 5% nonfat milk solution in Tris-buffered saline with 0.05% Tween 20 (TBST) for 1 h at room temperature and then incubated overnight at 4°C with the appropriate primary antibody (RNF146 1:1000; LC3II 1:1500; Beclin-1 1:1000; P62 1:2000; β-actin 1:2500; β-catenin 1:500). The membranes were washed in TBST and incubated for 1 h at room temperature with the secondary antibodies diluted in blocking buffer. Immunoreactivity was detected by SuperSignal West Pico Chemiluminescent Substrate (Thermos Scientific). The optical densities of the bands were quantified by using an image analysis system with ImageJ (Scion Corporation, Torrance, CA, USA).

### Immunofluorescence

After incubated with 4% paraformaldehyde for 15 min at 37°C, HT22 cells were rinsed with PBS for three times and permeabilized with 0.2% Triton X-100 for 10 min, followed by incubation with primary antibodies overnight at 40°C. The primary antibodies for immunohistochemistry were diluted as follows: RNF146 (1:50), LC3 (1:100) and β-catenin (1:100). Then, the cells were incubated with secondary antibodies (Alexa 488 donkey-anti-goat IgG, 1:300; Alexa-594-conjugated goat-anti-mouse IgG, 1:300) for 2 h. Cultures were dehydrated with ethanol and mounted with 4′6-diamidino-2-phenylindole (DAPI) 10 min for nuclear staining (Sigma). Images were captured by means of an Olympus FV10i Confocal Microscope (Olympus, Tokyo, Japan). All images from one experiment were acquired using the same exposure time to allow comparisons of relative levels of immunoreactivity between the different treatment conditions. At least six images of each group were taken by an evaluator that was blinded to the experimental conditions.

### Lentivirus Construction and Transfection

Both the lentivirus RNF146 and shRNA of RNF146 were purchased from ShanghaiGeneChem. The lentivirus sequence of RNF146 was amplified by RT-PCR. In brief, the sequences of RNF146 cDNA were as follows: 5′-GAGGATCCCCGGGTACCGGTCGCCACCATGGAGATGGCCGGCTGTG-3′ and 5′-TCATCCTTGTAGTCGCTAACCTCTGTCACTGTGCACTGC-3′. To generate the recombinant lentivirus expressinhg-RNF146 (LV-RNF146), 293T cells were co-transfected with the Ubi-MCS-3FLAG plasmid (ShanghaiGeneChem; 20 μg) with a cDNA that encoded for RNF146, pHelper1.0 plasmid (15 μg), and pHelper 2.0 plasmid (10 μg) by using Lipofectamine 2000 (Invitrogen; 100 μl). After 48 h, the supernatant was harvested from the cultures, and the viral titer was calculated by transducing 293T cells. As a control, we also generated a control lentiviral vector that expresses GFP alone (LV-NC). The lentivirus vectors encoding short hairpin RNA targeting mouse-RNF146 (si-RNF146) was obtained from GV118 under the control of CMV promotor. Target sequences of RNF146-RNAi are as follows: 5′-GTGACACCAATACTGTAAAT-3′. GV118 containing scrambled sequence was used as the control plasmid to produce negative control shRNA (si-NC). Cells were transfected with lentivirus vectors for 48 h depending on manufacturer’s instructions and subjected to various treatments.

### Cell Viability Assay

A cell viability assay was conducted using the MTT cell proliferation assay kit (Sigma-Aldrich). Cells were seeded in 96-well plates at densities of 5000 cells per well, and 0.5 mg/ml of MTT was added to each well. The plate was incubated at 37°C for 4 h. Then, the supernatant-containing MTT was removed, and 200 μl of DMSO was added to each well. The plate was shaken thoroughly for 1 min on a shaker. The absorbance of the samples was measured at 570 nm by using a microplate reader (Bio-Rad Laboratories, Hercules, CA, USA).

### Lactate Dehydrogenase (LDH) Assay

Cytotoxicity was determined by the release of Lactate dehydrogenase (LDH), a cytoplasmic enzyme that is released from cells and that indicates membrane integrity. LDH release into the culture medium was detected using a diagnostic kit according to the manufacturer’s instructions. Briefly, 50 μl of supernatant from each well was collected to assay the LDH release. The samples were incubated with the reduced form of nicotinamide-adenine dinucleotide (NADH) and pyruvate for 15 min at 37°C, and the reaction was stopped by adding 0.4 mol/l NaOH. The LDH activity was calculated from the absorbance at 440 nm, and the background absorbance from culture medium that was not used for any cell cultures was subtracted from all absorbance measurements. The results were normalized to the maximal LDH release, which was determined by treating control wells with 1% Triton X-100 for 60 min to lyse all cells.

### Statistical Analysis

We applied at least five Samples for each Experiment. Statistical evaluation was conducted using the GraphPad Prism software, version 6.0 (GraphPad, San Diego, CA, USA). Significant differences between experiments were assessed by univariate analysis of variance (ANOVA; more than two groups), followed by Bonferroni’s multiple comparisons or unpaired *t*-test (two groups).

## Results

### RNF146 Exerted Protective Effects on Neuronal Cells after Glutamate-Induced Excitotoxicity

**Figure 1:**
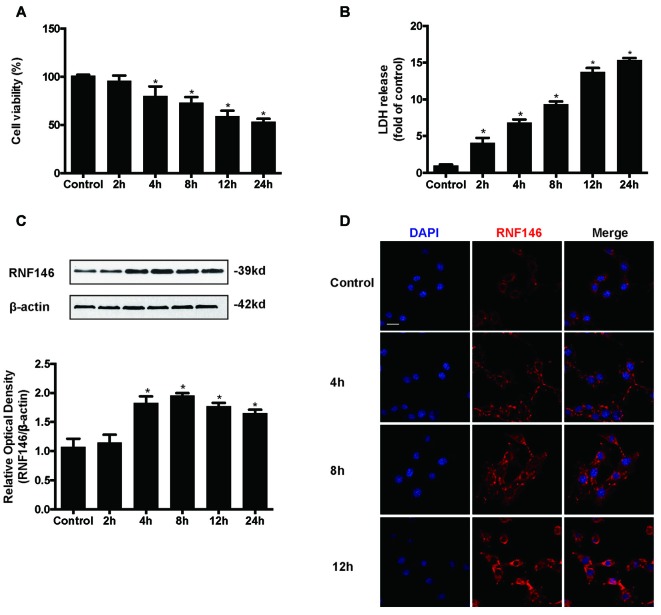
**RNF146 is protective in HT22 cells.** HT22 cells were treated with glutamate (10 mM) for 24 h. Cell viability was measured by MTT assay **(A)**, and cytotoxicity was measured by Lactate dehydrogenase (LDH) assay **(B)**. *P < 0.05 vs. The expression of RNF146 was detected by western blot analysis **(C)**. The data are represented as the mean ± SEM. from five experiments. *P < 0.05 vs. control. The expression of RNF146 protein in different time points was examined by Immunofluorescence staining **(D)**. The nuclei were stained by 4′6-diamidino-2-phenylindole (DAPI). Scale bar = 20 μm.

HT22 cells were incubated in the presence of glutamate (10 mM) for various lengths of time (2 h, 4 h, 8 h, 12 h and 24 h). Assessment of cell viability and LDH release indicated that glutamate-induced nearly 50% of the cell death at 24 h (Figures 1A,B). The expression levels of RNF146 were detected by Western blot and immunofluorescent staining (Figures 1C,D). RNF146 levels were elevated in HT22 cells after incubated with glutamate for 4 h, being maximal at 8 h.

**Figure 2:**
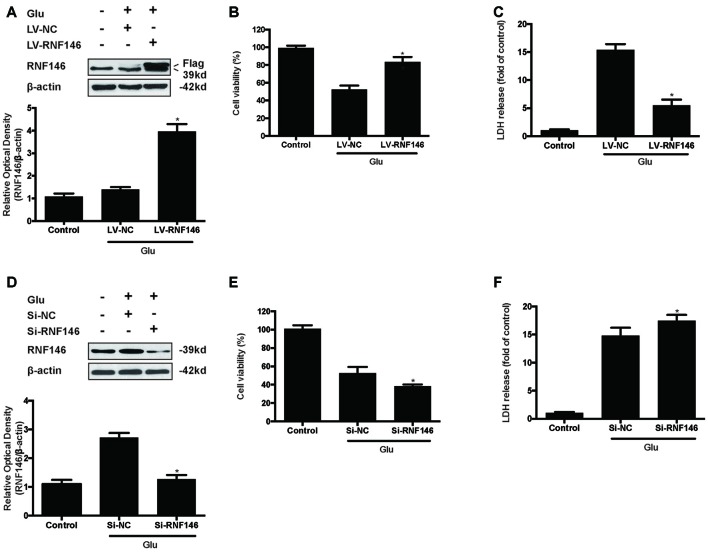
**RNF146 protects HT22 cells during glutamate excitotoxicity.** HT22 cells were infected with LV-RNF146 for 24 h and cultured for another 48 h then exposure to glutamate (10 mM) for 24 h. Glu is the abbreviation of glutamate, unless otherwise specified. RNF146 protein level was detected by Western blot after treated with or without glutamate **(A)**. The data are represented as the mean ± SEM. from five experiments. *P < 0.05 vs. negative control. Cell viability and LDH release were measured in presence or absence of glutamate **(B,C)**. The data are represented as the mean ± SEM. from five experiments. *P < 0.05 vs. LV-NC group. Si-RNA of RNF146 transfected HT22 cells for 24 h and cultured for another 48 h then treated with glutamate. The expression of RNF146 was identified by Western blot analysis **(D)**. Cell viability and cytotoxicity were measured in HT22 cells after glutamate excitotoxicity **(E,F)**. Data are expressed as mean ± SD of five independent experiments. *P < 0.05 vs. vector.

To identify the effect of RNF146 on glutamate-induced excitotoxicity, HT22 cells were transfected with LV-RNF146 or LV-negative control for 4 h. After media replacement and 48 h further incubation, cells were treated with glutamate for 24 h. Following transfection and glutamate treatment, lentiviral transduction of LV-RNF146 improved the expression of RNF146 protein (Figure 2A), which inhibited the reduction of cell viability (Figure 2B) and decreased the release of LDH (Figure 2C) in HT22 cells. To further elucidate the effects of endogenous RNF146 on glutamate excitotoxicity, the expression of RNF146 protein was knocked down by a lentivirally expressed siRNA that targeted RNF146 (si-RNF146; Figure 2D). Knock-down of RNF146 increased the susceptibility of the cells to glutamate excitotoxicity leading to the decreasing of cell viability (Figure 2E) and increasing of LDH release (Figure 2F). In addition, this protective effects of RNF146 against glutamate excitoxic injury were also observed in primary neuron cultures (Supplementary Figure 1). Taken together, these results indicated that RNF146 might be an endogenous neuroprotective protein during glutamate excitotoxicity.

### RNF146 Reduced the Autophagy in Neuronal Cells Undergoing Glutamate Excitotoxicity

**Figure 3:**
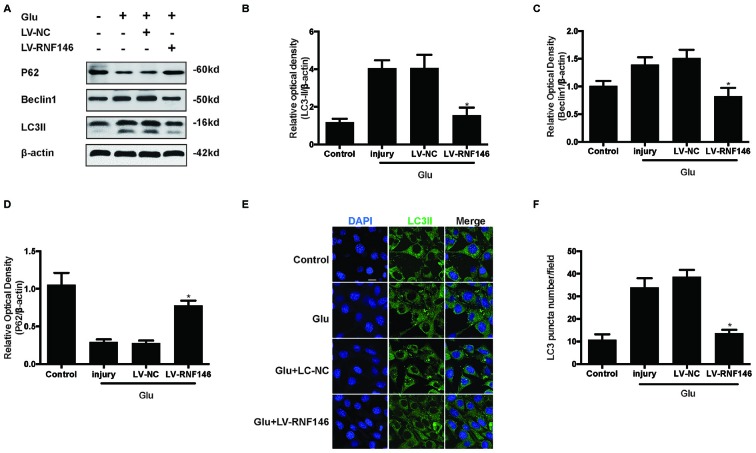
**Upregulating of RNF146 attenuated autophagy in HT22 cells.** Similarly, HT22 cells were transfected with LV-RNF146 and cultured for 2 days. The cells were treated with glutamate (10 mM) for 24 h then applied to western blot and Immunofluorescence analysis. The expression of LC3II, P62 and Beclin1 were detected by western blot **(A–D)**. The data are represented as the mean ± SEM. from five experiments. *P < 0.05 vs. negative control. Immunofluorescence staining was used to assess the effect of over-expression of RNF146 on autophagy in HT22 cells **(E)**. Nuclei were strained with DAPI. Scale bar = 20 μm. Quantification of LC3-positve puncta were assessed at 24 h after glutamate excitotoxicity **(F)**. Values are mean ± SEM from five experiments. *P < 0.05 vs. negative control.

**Figure 4:**
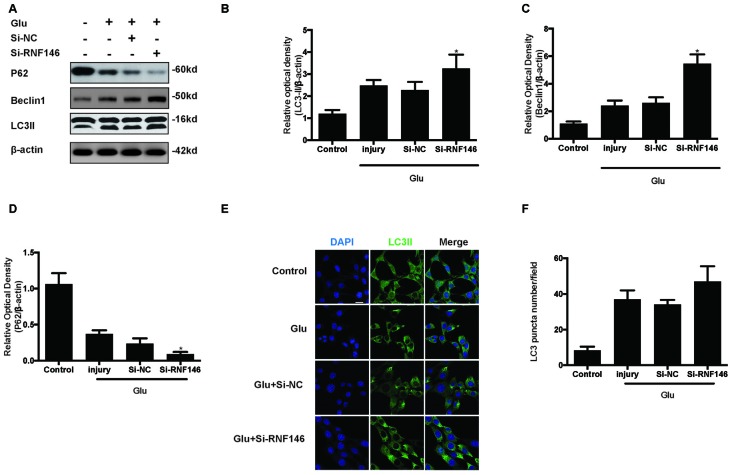
**Reduction of RNF146 promoted autophagy in HT22 cells.** HT22 cells were transfected with Si-RNF146 or Si-NC before undergoing glutamate excitotoxicity. Likewise, the expression of LC3II, P62 and Beclin1 at 24 h after exposure to glutamate (10 mM) was assessed by western blot analysis **(A–D)**. The data are represented as the mean ± SEM. from five experiments. *P < 0.05 vs. negative control. LC3II was also detected with Immunofluorescence staining at 24 h after glutamate excitotoxicity in differently treated HT22 cells **(E)**. Nuclei were strained with DAPI. Scale bar = 20 μm. Quantification of LC3-positve puncta were assessed at 24 h after glutamate excitotoxicity **(F)**. Values are mean ± SEM from five experiments.

To examine whether the regulation of RNF146 could affect autophagy following glutamate excitotoxicity, we transfected HT22 cells with LV-RNF146 and then cultured the cells for 2 days before adding glutamate. Our results indicated that the effect of the overexpression of RNF146 decreased protein levels of LC3II and beclin-1 and increased the expression of p62 (Figures 3A–D). The immunofluorescent staining results showed that overexpression of RNF146 significantly diminished the LC3II puncta (Figures 3E,F). Similar results were collected from studies in primary neuron cultures (Supplementary Figure 1). To further clarify the effects of RNF146 on regulating autophagy after glutamate excitotoxicity, RNF146 protein was knocked down by si-RNF146. We found that knockdown of RNF146 increased the expression of LC3II and beclin-1 and decreased the p62 protein level (Figures 4A–D). However, it is not obviously determined by immunofluorescent staining (Figures 4E,F).

### RNF146 Inhibited Glutamate-Induced Excitotoxicity by Negatively Regulating Autophagy

**Figure 5:**
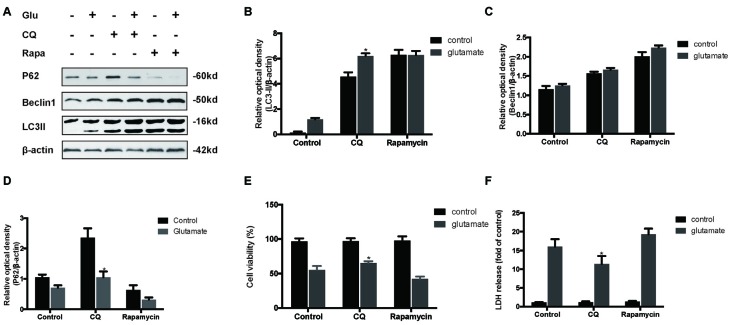
**Inhibition of autophagy attenuated the HT22 cells damage after glutamate-induced excitotoxicity.** We co-cultured transfected HT22 cells with Rapamycin (Rapa) or chloroquine (CQ) for 24 h then glutamate applied for 24 h. LC3II, P62 and Beclin1 were then examined by western blot analysis **(A–D)**. The data are represented as the mean ± SEM. from five experiments. *P < 0.05 vs. control. Cell viability and cytotoxicity were measured in HT22 cells after glutamate excitotoxicity **(E,F)**. Data are expressed as mean ± SEM of five independent experiments. *P < 0.05 vs. control.

To determine the role of autophagy in glutamate excitotoxicity, we assessed the extent of cell injury after HT22 cells undergoing glutamate and pharmacological agents by which regulated autophagy (Figures 5A–D). The results showed that rapamycin (Rapa, 4 μM), a classical activator of autophagy, aggravated excitotoxicity in HT22 cells, thereby proving that over-activated autophagy is detrimental to HT22 cells during glutamate excitotoxicity (Figures 5E,F). Moreover, we also compared rapamycin with CQ (10 μM), an inhibitor of autophagy, to detect their autophagy-regulating effects on the glutamate-induced injury. The results indicated that CQ inhibited uncontrolled autophagy and was beneficial to HT22 cell survival (Figures 5E,F).

**Figure 6:**
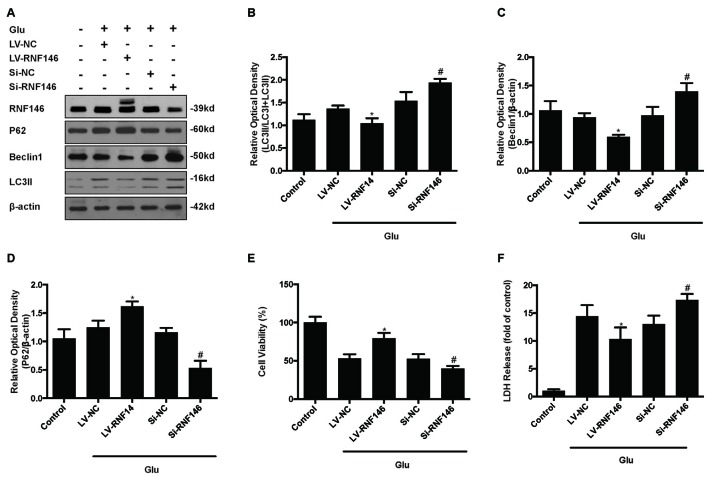
**Regulation of RNF146 modulated the glutamate-induced excitotoxicity damage via affecting autophagy.** HT22 cells were pretreated with LV-RNF146 or Si-RNF146 before adding glutamate or control. The expression of RNF146, P62, Beclin1 and LC3II were detected by western blot analysis **(A–D)**. Cell viability and cytotoxicity were measured in HT22 cells after glutamate excitotoxicity **(E,F)**. The data are represented as the mean ± SEM from five experiments. *P < 0.05 vs. LV-NC. ^#^*P* < 0.05 vs. Si-NC.

**Figure 7:**
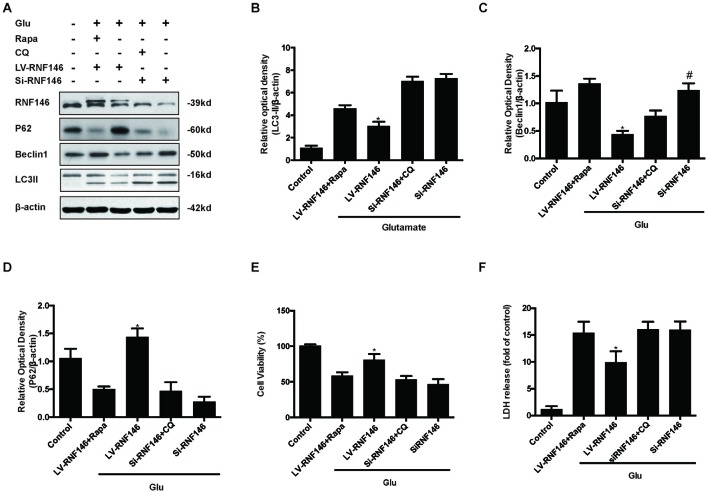
**Rapamycin blocked the protective function of RNF146.** HT22 cells exposure to Rapamycin (Rapa) or CQ for 24 h after transfection. The level of RNF146, P62, Beclin1 and LC3II were detected by western blot **(A–D)**. Cell viability and cytotoxicity were measured in HT22 cells after glutamate excitotoxicity **(E,F)**. The data are represented as the mean ± SEM from five experiments. *P < 0.05 vs. LV-RNF146 + Rapa. ^#^*P* < 0.05 vs. Si-RNF146 + CQ.

Since previous results showed that RNF146 exerted a neuroprotective effect and negatively regulates autophagy after glutamate excitotoxicity, we assumed that RNF146 influenced excitotoxicity via modulation of autophagy. LV-RNF146 transfection into HT22 cells was used to detect its specific roles in autophagy-related injury. After administration of glutamate for 24 h, the up-regulation of RNF146 promoted cell viability and reduced the degree of neurotoxicity by suppressing induction of autophagy, while cell injury deteriorated by down-regulation of RNF146 and its role in enhancement of autophagy (Figure 6). Moreover, the protective effects of RNF146 were reversed by rapamycin treatment during glutamate excitotoxicity injury. Correspondingly, down-regulation of RNF146 had a positive effect on stimulating autophagy, thereby resulting in cell death. However, the function of si-RNF146 transfection was not significantly reversed by CQ during glutamate excitotoxicity. The cell viability between si-RNF146 transfection and si-RNA transfection with CQ showed no obvious difference (Figure 7).

### RNF146 Induced the Facilitation of Wnt/β-Catenin Pathway after Glutamate-Induced Excitotoxicity

**Figure 8:**
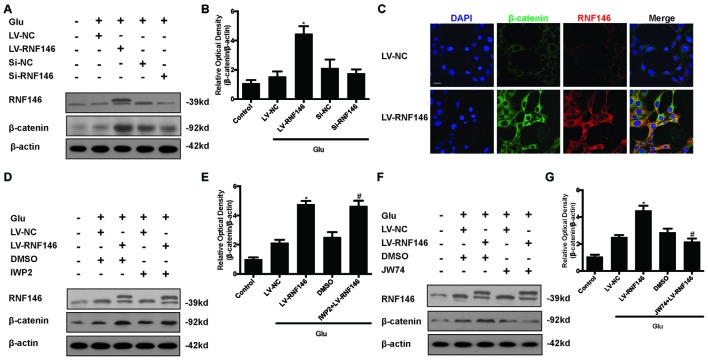
**Wnt/β-catenin pathway is affected by alteration of RNF146.** After transfected with LV-RNF146 or Si-RNF146, Western blot was used to analysis the level of RNF146 and β-catenin after HT22 cells exposure to glutamate for 24 h **(A,B)**. *P < 0.05 vs. LV-NC. Double stating was utilized to illustrate their connection between RNF146 and β-catenin **(C)**. Scale bar = 20. Two type of Wnt inhibitors (IWP2 and JW74) were also used to detected the exquisite site of RNF146 function. RNF146 and β-catenin were detected by western blot analysis **(D–G)**. *P < 0.05 vs. LV-NC. ^#^*P* < 0.05 vs. DMSO. The data are represented as the mean ± SEM from five experiments.

To investigate the relationship between RNF146 and Wnt signaling in glutamate excitotoxicity, HT22 cells were treated with LV-RNF146 for 24 h and then cultured 2 days before other treatments. After treatment with glutamate for 24 h, overexpression of RNF146 stimulated the elevation of β-catenin expression when compared with the control group. The down-regulation of RNF146 by siRNA followed by a decrease in β-catenin (Figures 8A,B). Further immunofluorescence staining of RNF146 and β-catenin indicated that the upregulation of RNF146 was related to β-catenin overexpression (Figure 8C). Taken together, RNF146 is a positive regulator of Wnt/β-catenin, the downstream modulator of Wnt signaling, during glutamate-induced excitotoxicity.

To further elucidate the targets of RNF146 in Wnt/β-catenin signaling, we used inhibitors to interfere the different processes of Wnt/β-catenin signaling transduction. Changes of β-catenin induced by up-regulation of RNF146 was not affected via using the IWP2 (10 μM), an inhibitor of Wnt processing and secretion (Figures 8D,E). However, JW74 (10 μM), as a promotor of axin2 stabilization, blocked the effects of RNF146 on the regulation of β-catenin (Figures 8F,G). These results indicated that positive modulation of Wnt/β-catenin pathway by RNF146 was tightly related to regulation of axin2 and its interaction with β-catenin.

### RNF146 Attenuated Autophagy via Regulating Wnt/β-Catenin Pathway

**Figure 9:**
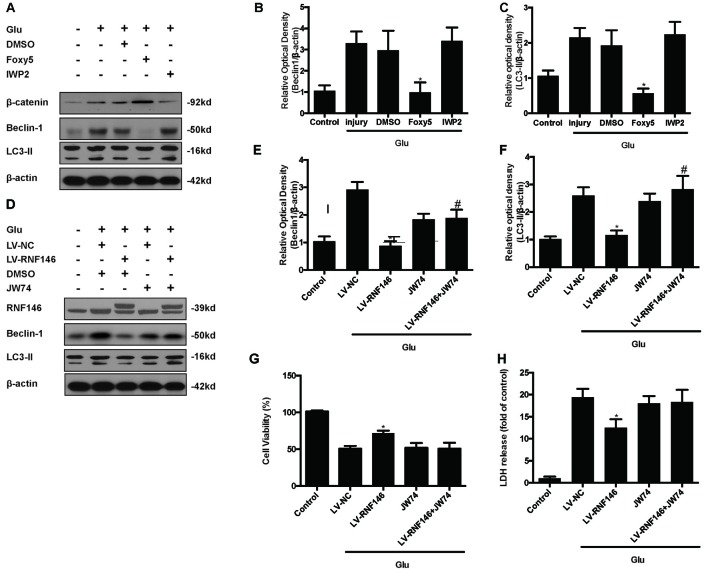
**Inhibitor of Wnt/β-catenin pathway reversed the RNF146 inactivation of autophagy.** Wnt/β-catenin pathway agonist Foxy5 and inhibitor IWP2 were used to examined its function after glutamate excitotoxicity. Western blot was used to analysis the level of β-catenin, Beclin1 and LC3II **(A–C)**. *P < 0.05 vs. DMSO. Furthermore, we applied JW74 to detect whether inhibit Wnt/β-catenin pathway could reverse the function of LV-RNF146. Western blot was used to analysis the level of RNF146, Beclin1 and LC3II **(D–F)**. *P < 0.05 vs. LV-NC. ^#^*P* < 0.05 vs. JW74. Cell viability and cytotoxicity were measured in HT22 cells after glutamate excitotoxicity **(G,H)**. The data are represented as the mean ± SEM from five experiments. *P < 0.05 vs. LV-NC.

To identify the mechanism by which RNF146 modulates autophagy, we first applied Foxy5, a Wnt5a peptide mimetic, and antagonist IWP2 to examine their function in autophagy. β-catenin levels were increased after treatment with Foxy5 in cells undergoing glutamate excitotoxicity. When compared with the non-Foxy5-treated group, the Foxy5-treated group decreased levels of the autophagy-related protein LC3II and beclin-1. Correspondingly, β-catenin levels were decreased by IWP2, followed by an increase in LC3II and beclin-1 protein levels following glutamate excitotoxicity (Figures 9A–C).

To examine whether the Wnt/β-catenin pathway is involved in the negative regulation of autophagy by RNF146, we used the Wnt/β-catenin signaling inhibitor JW74. HT22 cells were transfected with LV-RNF146 for 48 h before being exposed to glutamate excitotoxicity. JW74 were administered 2 h prior to the injury. JW74 reversed the overexpression of RNF146-induced inactivation of autophagy, and then worsen the cell injury after glutamate treatment (Figures 9D–H). This result modulation of autophagy by RNF146 was related to its regulation of Wnt/β-catenin signaling.

## Discussion

RNF146 has a protective function in DNA damage-induced cell death (Kang et al., 2011) and oxidative cell death (Gerö et al., 2014), depending on its ubiquitin E3 ligase activity. Previous studies have indicated that RNF146’s protective effects are dependent on interfering with parthanatos by modulating PARP-1 and apoptosis inducing factor (AIF; Andrabi et al., 2011; Xu et al., 2013). RNF146 blocks the translocation of AIF from the mitochondria to the nucleus by activating PARP-1. In the present study, we demonstrated that RNF146 increased in a time-dependent manner after glutamate was applied to HT22 cells. Overexpression of RNF146 attenuated cell injury that was induced by glutamate. Conversely, interfering with RNF146 expression produced cell damage. Our findings indicated that the activation of RNF146 is a self-protective mechanism in HT22 cells.

Autophagy is a key adaptive response to micro-environmental stress and a vital mechanism for recycling cellular components to sustain viability when cells have outstripped their exogenous nutrient supply (Kroemer et al., 2010). In neurons, autophagy normally preserves normal material metabolism and protects cells under stress from nutrient deprivation, energy loss, or states of protein aggregation by breaking down less essential cellular constituents (Hara et al., 2006; Komatsu et al., 2006). Although autophagy is generally neuroprotective, excessive activity is deleterious. Regarding autophagy tightly related to autophagic cell death (ACD), some groups have reported that the over-stimulated activity of autophagy can contribute to lethality (Shi et al., 2012; Chen et al., 2015). Some of the strongest support for ACD as a pathological process has been found under conditions of excitotoxicity and hypoxia-ischemia in central nervous system, which produce stress that may be the result of trauma or stroke (Balduini et al., 2009; Chen et al., 2014). Inhibiting autophagy has been reported to be beneficial in several neurodegenerative states (Wen et al., 2008; Xing et al., 2012). Similarly, this cell death is reduced by the inhibition of major signaling in autophagosome formation by using the PI3K inhibitor 3-MA, or via genetic knockdown of Atg7 and beclin-1(Koike et al., 2008; Puyal et al., 2009). In this study, our results also supported that over-stimulated autophagy during glutamate-induced excitotoxicity decreased cell viability. Taken together, autophagy is required to maintain physiological homeostasis, while during the pathological condition over-activated autophagy is detrimental to cell status.

According to our results, RNF146 is a critical regulator of autophagy progress under glutamate excitotoxicity. RNF146 contains two structural domains, a RING finger domain, which is predicted to interact with an E2, and a WWE domain, which is predicted to interact with PARsylated proteins. Previous study suggested that the RNF146 WWE domain cannot recognize ADPR, but can specifically identify iso-ADPR moiety of PAR (DaRosa et al., 2015). The interaction between the WWE domain and PAR is involved in the RNF146-dependent regulation of axin *in vivo*. Ring finger domain is the functional module for E3 ubiquitin ligase activity, which activates a ubiquitin-conjugating enzyme (E2) to transfer ubiquitin directly from the E2 active site to a lysine residue of a substrate (DaRosa et al., 2015). As a new regulator of Wnt signaling, RNF146 promotes the Wnt pathway by repressing degradation of β-catenin in HEK293 cell lines (Callow et al., 2011). β-catenin is an essential transcriptional co-regulator in the canonical Wnt pathway and is associated with tumor proliferation and a variety of diseases. It is also known to facilitate adaptation to micro-environmental conditions, including hypoxia (Kaidi et al., 2007) and oxidative stress (Tao et al., 2013). However, the critical role of RNF146 in controlling β-catenin and Wnt signaling following glutamate excitotoxicity is not well understood. Here, our results indicated that overexpression of RNF146 enhanced β-catenin protein levels, while lower RNF146 levels decreased β-catenin protein levels after glutamate-induced excitotoxicity, thereby indicating that the regulation of Wnt signaling by RNF146 has an important role in glutamate-induced excitotoxicity.

Previously, the role of the Wnt/β-catenin pathway in regulating autophagy was controversial. Previous studies have proven that β-catenin negatively regulates autophagy in vascular calcification and prostate cancer (Liu et al., 2014; Lin et al., 2015). The inhibition of β-catenin significantly increased LC3II expression and induced ACD. The increase of β-catenin signaling reduced beclin-1 expression and rescued endothelial cells from endostatin-induced autophagy. In contrast, β-catenin has a positive role in modulating autophagy in matrine-induced autophagy (Xie et al., 2014). Due to this contradictory results, it is difficult to illustrate the accurate function of β-catenin in regulating autophagy. Although these experiments were not conducted in the same models of diseases, our results agree with the former results. It is known that the Wnt/β-catenin pathway is closely connected to RNF146 and autophagy. We explored the role of RNF146 in regulating the Wnt/β-catenin pathway in neuroprotection and negatively regulating autophagy. Our results indicate that overexpression of RNF146 stimulated the Wnt/β-catenin pathway and reduced the autophagy rate, thereby reducing the effects of glutamate excitotoxicity. To gain further insights into the mechanism of RNF146-Wnt/β-catenin-autophagy pathway, we used two Wnt signaling inhibitors IWP2 and JW74. IWP2 suppresses the Wnt processing and secretion, while JW74 enhances the expression of axin2 and the stabilization of β-catenin destruction complex (Chen et al., 2009; Stratford et al., 2014). Although IWP2 did not affect the role of RNF146 in regulating Wnt signaling, JW74 reversed the promoting effects that were induced by RNF146 in the Wnt/β-catenin pathway, which disabled the effects of RNF146 on inhibiting autophagy and neuroprotection. Thus, modulation of β-catenin destruction complex should be the major mechanism underlying RNF146-Wnt/β-catenin-autophagy pathway following glutamate-induced excitotoxicity.

Glutamate excitotoxicity is a common occurrence following brain injury and other neurological diseases. Our previous study proved that overexpression of RNF146 could attenuate H_2_O_2_ induced mitochondria dysfunction in HT22 cells. However, RNF146 had no effect on cytochrome c release, elevation of Bax/Bcl-2 ratio and cleavage of Caspase-9, and activation of Caspase-3, which indicated RNF146 did not affect the mitochondria-associated apoptosis (Xu et al., 2013). According to our study, RNF146 can attenuate excessive autophagy that is induced by glutamate excitotoxicity, thereby improving cell viability, which provides a new mechanism for the neuroprotection role of RNF146. Nevertheless, little is known about the underlying signaling events that are responsible for the ability of RNF146 to modulate autophagy activity. Although we have demonstrated that RNF146 regulates autophagy via the Wnt/β-catenin pathway, the E3 ubiquitin ligase RNF146 may possess other direct relationships with autophagy. Further investigations are required for a better understanding of the underlying mechanism of the RNF146/autophagy pathway and its significance in cell survival after TBI, stroke or other types of neurological diseases.

## Author Contributions

YY, PL and HaX helped fulfill the western blot experiments and data statistical analysis. SD, WR and CP assisted to do Immunofluorescence staining. WM, JW and HoX helped to analyze the immunofluorescence results. LZ, SZ and ZF helped to revise the manuscript.

## Funding

The work was supported by National Natural Science Foundation of China (Nos. 81430043, 30930093, 81301037, 81601077), the National Science and Technology Major Project of China (2013ZX09J13109-02C), the National Science and Technology Pillar Program of China (No. 2012BAI11B02), the Science and Technology Project of Shaanxi (No. 2013KTCQ03-01), the Program for Changjiang Scholars and Innovative Research Team in University (No. IRT-14208), and the Key Project of the Twelfth Five-year Plan of Scientific Research of China (AWS11J008).

## Conflict of Interest Statement

The authors declare that the research was conducted in the absence of any commercial or financial relationships that could be construed as a potential conflict of interest.
