# Supplementary material for: RNF146 Inhibits Excessive Autophagy by Modulating the Wnt-β-Catenin Pathway in Glutamate Excitotoxicity Injury
Source: Front Cell Neurosci. 2017 Mar 6;11:59. doi: 10.3389/fncel.2017.00059 (PMC5337692; doi:10.3389/fncel.2017.00059)
Supplement: Supplementary file 1 [file DataSheet_1.docx]

Supplementary Material

RNF146 inhibits excessive autophagy by modulating the Wnt-β-catenin pathway in glutamate excitotoxicity injury

**Yuefan Yang^1,a^, Peng Luo^1,a^, Haoxiang Xu^3,4,a^, Shuhui Dai^1^, Wei Rao^1^, Cheng Peng^1^, Wenke Ma^1^, Jiu Wang^1^, Hongyu Xu^1^, Lei Zhang^1^, Sai Zhang^2^, Zhou Fei^1,*^.**

*** Correspondence:** Zhou Fei: [zhoufei@fmmu.edu.cn](mailto:zhoufei@fmmu.edu.cn)

# Supplementary Data

Primary neuron culture was prepared from embryology 18 day C57BL/6 mouse. In short, Poly-L-lysine were used to coat surface for at least 3 hours at 37°C and washed with double distilled water. Cerebral hemispheres were carefully dissociated from embryos and separated from meninges in pre-cold DMEM. Cortex tissues were transferred to a new tube and applied to digest with trypsin (0.5%) for 20 min. DMEM containing 10% fetal bovine serum were applied to terminate the digestion. The cells were added Dnase for 2 min to guarantee the harvest, then centrifuged two times at 1000rpm for 5min to remove the Dnase enzyme and trypsin. The neuron cells were plated on pre-coated plates at with neurobasal medium containing B27 and L-glutamine. Cells were cultured for 8 days before proceeding transfection.

## Supplementary Figures


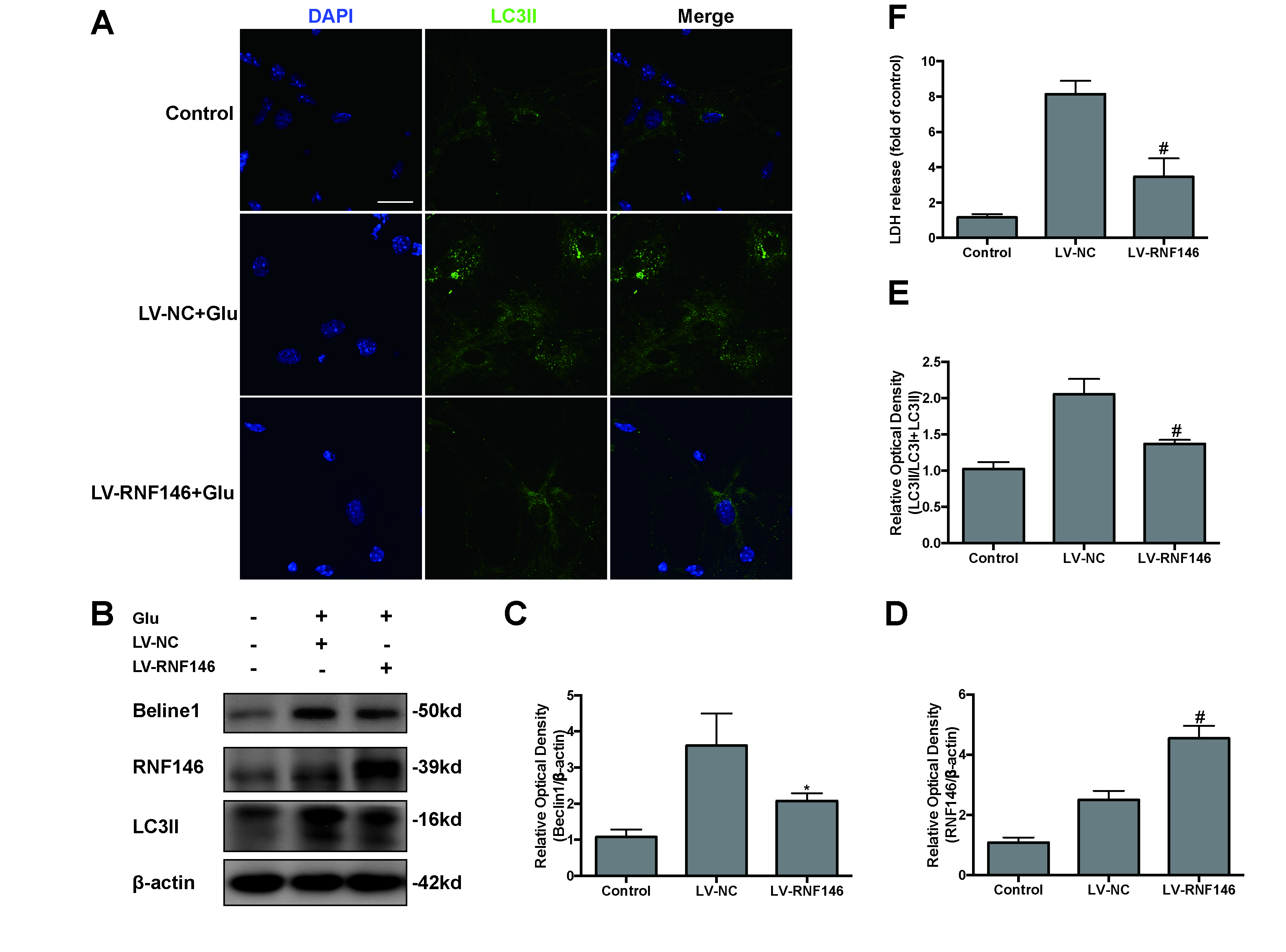


**Supplementary Figure 1.** Upregulating of RNF146 RNF146 attenuates the expression of autophagy and protects the neurons. Primary cultured neurons were transfected with LV-RNF146 or LV-NC for 48 h then exposure to glutamate (5mM) for 24 h. Immunofluorescence staining were used to detect LC3 pattern after glutamate treatment (**A**). The expression of LC3II, RNF146 and Beclin1 were detected by western blot (**B-E**). The data are represented as the mean+S.E.M. from four experiments. ^*^*P*<0.05 *versus* negative control. ^#^*P*<0.01 *versus* negative control. Cell cytotoxicity were measured in neurons after glutamate excitotoxicity(F). The data are represented as the mean+S.E.M. from four experiments. ^#^*P*<0.01 *versus* negative control.
